# Pressure effects on lipids and bio-membrane assemblies

**DOI:** 10.1107/S2052252514019551

**Published:** 2014-09-23

**Authors:** Nicholas J. Brooks

**Affiliations:** a Department of Chemistry, Imperial College London, South Kensington Campus, London SW7 2AZ, England

**Keywords:** biological membranes, lipids, bilayers, lipid–protein assemblies, high-pressure studies

## Abstract

Membranes are amongst the most important biological structures; they maintain the fundamental integrity of cells, compartmentalize regions within them and play an active role in a wide range of cellular processes. Pressure can play a key role in probing the structure and dynamics of membrane assemblies, and is also critical to the biology and adaptation of deep-sea organisms. This article presents an overview of the effect of pressure on the mesostructure of lipid membranes, bilayer organization and lipid–protein assemblies. It also summarizes recent developments in high-pressure structural instrumentation suitable for experiments on membranes.

## Abstract 2

Pressure can play a key role in probing the structure and dynamics of membrane assemblies, and is also critical to the biology and adaptation of deep-sea organisms. This article presents an overview of the effect of pressure on the structure of membranes and recent developments in high-pressure instrumentation.

## 1. Introduction   

Over 70% of the Earth is covered with water, to an average depth of 3.8 km, which exerts a pressure of almost 40 MPa (400 bar). Despite such high pressures, life thrives in the ocean. Indeed, over the past 30 years, pressure-adapted organisms have been discovered in increasingly extreme ocean conditions. The bottom of the Marianas Trench reaches 11 km below sea level, exerting a pressure greater than 100 MPa (1 kbar) (Picard & Daniel, 2013 ▶), and even at these pressures adapted bacteria have been found. These organisms must have mechanisms to adapt their lipid membranes to maintain their fundamental structure and mechanical properties (Bartlett, 2002 ▶). While high-pressure adaptation is a well established phenomenon (Meersman & McMillan, 2014 ▶; Casadei *et al.*, 2002 ▶), the regulatory mechanisms employed are poorly understood and are the subject of great interest.

In addition to its direct relevance to the biology of deep-sea organisms, high pressure can play a key role in studying the structure and dynamics of biological assemblies. Hydrostatic pressure can be used to drive structural changes in bio­­mol­ecules, and offers significant advantages over other structure-change triggers such as temperature and composition changes, both at equilibrium and during rapid changes: high pressure does not tend to disrupt intramolecular bonding below 2 GPa; pressure can be applied and released from a sample extremely rapidly with both increasing and decreasing pressure; and, due to the rapid propagation of pressure, it equilibrates throughout a sample quickly. Many structural changes in membrane assemblies take place on the millisecond to second timescale and so, by using fast pressure jumps, the thermodynamic trigger variable can be decoupled from the structural change, allowing the real-time kinetics and evolution of these changes to be studied using fast-probe techniques.

This review gives an overview of recent advances in high-pressure structural investigations of model membrane assemblies and of some of the recent technology developments that have underpinned these experiments.

## 2. Effect of pressure on membrane assemblies   

Membranes are amongst the most important of all biological structures. In addition to maintaining basic cell integrity and compartmentalization, lipids are known to play a vital role in cell signalling, and there is increasing evidence that the micromechanics of membranes help to modulate the activity of the proteins, peptides, channels and receptors embedded within them (van den Brink-van der Laan *et al.*, 2004 ▶).

**Figure 1:**
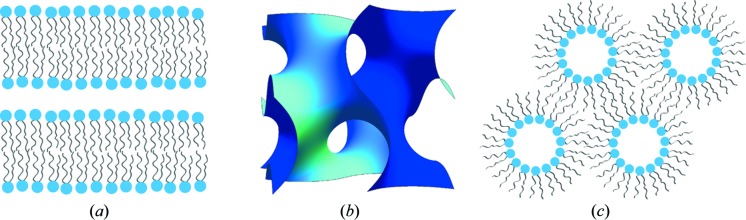
Examples of self-assembled lipid lyotropic liquid crystal structures. (*a*) Lamellar (L_α_), (*b*) gyroid inverse bicontinuous cubic (Q_II_^G^) (a bilayer is draped over the minimum surface is shown) and (*c*) inverse hexagonal (H_II_).

The structural role of lipid membranes in biology is underpinned by the fact that lipids are amphiphilic molecules and so can self-assemble. Lipids form a variety of type I (normal) and type II (inverse) lyotropic liquid crystalline phases when mixed with water (Seddon, 1990 ▶) (Fig. 1 ▶). These include the fluid lamellar (L_α_), two-dimensional hexagonal (H_I_/H_II_) and inverse bicontinuous cubic phases (Q_II_^G^, Q_II_^D^, Q_II_^P^), and ordered micellar phases (including a number of recently discovered novel ordered inverse micellar structures) (Shearman *et al.*, 2009 ▶; Perroni & Mahanthappa, 2013 ▶). The structure adopted by a hydrated lipid assembly depends strongly on the preferred curvature of the lipids, as well as more subtle effects such as the interplay between curvature, elastic stress and chain-packing frustration (Shearman *et al.*, 2006 ▶). All of these factors can be affected by pressure (Seddon *et al.*, 2006 ▶).

### 2.1. Membrane curvature   

The effect of pressure on any structure is to drive a reduction in volume (Royer, 1995 ▶) and, in lipid assemblies, the net result of increasing pressure is a reduction in hydrocarbon chain motion and a corresponding increase in chain ordering (Skanes *et al.*, 2006 ▶). These effects will tend to reduce the cross-sectional area of the lipid hydrocarbon tails. Importantly, the cross-sectional area of the lipid head groups is significantly less sensitive to pressure and so increasing pressure will tend to increase the spontaneous curvature of a lipid monolayer (driving curvature away from the aqueous environment). It should be noted that, for type II (inverse curvature) systems, increasing pressure will cause a reduction in the magnitude of the preferred negative curvature (Shearman *et al.*, 2006 ▶).

**Figure 2:**
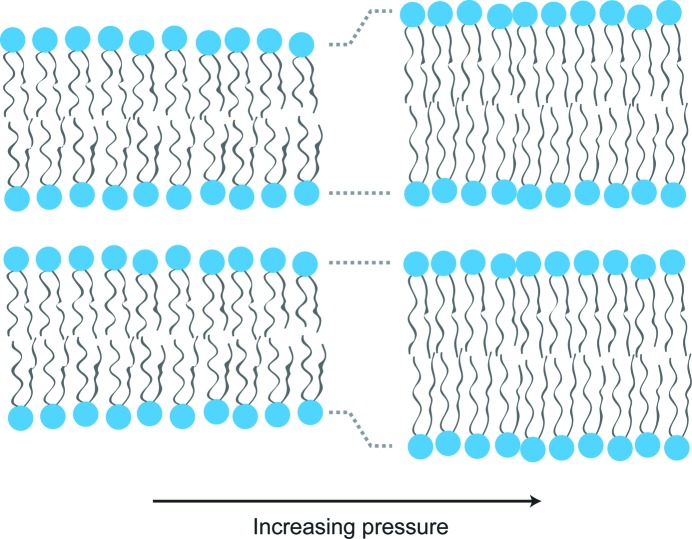
Pressure tends to increase chain ordering and chain extension, thereby increasing the thickness of flat bilayers.

**Figure 3:**
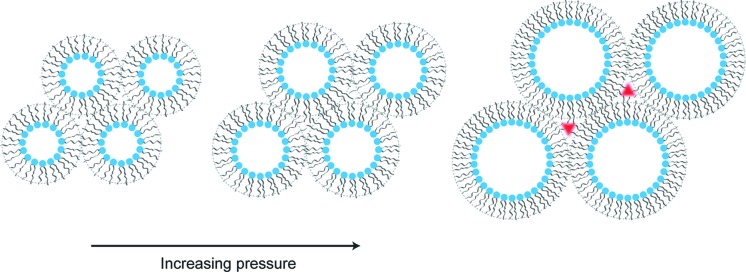
Swelling of an inverse hexagonal (H_II_) lipid phase. The chain-packing frustration increases as the diameter of the hexagonally packed cylindrical inverse micelles increases. This can be accommodated to a certain extent, but voids (shown in red) cannot be formed in the structure, thereby limiting the extent of pressure-induced swelling.

Moderate increases in pressure will tend to increase the lattice parameter of type 0 (flat) and type II (inverse) fluid lipid mesophases in contact with excess water. There are two distinct contributions to this effect. Firstly, chain ordering will tend to lead to an increase in the lattice parameter, as shown in Fig. 2 ▶, although this may be partially offset by isotropic compression of the water that is incorporated into the mesophase. Secondly, for inverse structures, increasing pressure will cause a reduction in the chain cross-sectional area, which will tend to reduce the magnitude of the negative curvature, leading to a significant increase in the lattice parameter (Fig. 3 ▶). This swelling mechanism relies on there being excess water available to flow into the swollen water channels and it is not observed under limited hydration conditions (Tang *et al.*, 2012 ▶). The inverse hexagonal H_II_ structure is formed from a hexagonal packing of cylindrical inverse micelles, which leads to chain-packing frustration (Fig. 3 ▶), where the lipid chains must adopt different conformations in different parts of the structure. The energy cost of this packing frustration increases at larger lattice parameters and so limits the pressure-induced swelling in H_II_ phases, though they still tend to swell slightly more than lamellar phases. Inverse bicontinuous cubic phases also suffer from packing frustration but to a much lesser extent than H_II_ structures (Seddon & Templer, 1993 ▶), and as a result they can swell by as much as 80 Å kbar^−1^ when subjected to high pressures (Winter *et al.*, 1999 ▶).

The effect of pressure on type I (normal curvature) structures is much more difficult to predict, as chain ordering will create a complex interplay between chain extension, which will tend to increase the lattice parameter, and a decrease in the lipid chain cross-sectional area, which will tend to increase the magnitude of the positive interfacial curvature and so reduce the lattice parameter. Very few high-pressure experiments have been carried out on type I curvature lyotropic liquid crystalline phases, but experimental results (Paccamiccio *et al.*, 2006 ▶) have demonstrated that pressure can induce a small but significant reduction in the lattice parameter of the *Ia*3*d* bicontinuous cubic two-dimensional hexagonal (H_I_) and *Pm*3*n* micellar cubic type I phases exhibited by hydrated dodecyltrimethylammonium chloride (DTAC). In all of these structures a change of around 0.5–1 Å kbar^−1^ was observed.

Over larger pressure ranges, pressure may also drive phase changes between lipid structures with significantly different interfacial curvature. This will occur when the pressure-induced change in the preferred curvature of the lipid mol­ecules is sufficient to make an alternative phase more energetically favourable. As described above, increasing pressure tends to increase the preferred interfacial curvature for a lipid monolayer and so, for type I systems, pressure will drive phase transitions to structures with larger interfacial curvature (*e.g.* lamellar to H_I_). Conversely, type II systems have a negative interfacial curvature, and so pressure drives transitions to structures with a smaller magnitude (more positive) curvature (*e.g.* H_II_ to lamellar).

Pressure has been seen to drive phase transformations in DTAC from a type I bicontinuous cubic to an H_I_ structure, and from the H_I_ phase to a *Pm*3*n* micellar cubic structure (Paccamiccio *et al.*, 2006 ▶); in both cases, pressure induces a phase transition to a structure with a higher type I curvature.

Pressure-induced phase changes in type II lipid systems have been studied far more widely than type I examples. Pressure has been observed to drive phase transitions between a wide range of type II structures, with pressure always causing a reduction in the magnitude of the interfacial curvature (Tyler *et al.*, 2011 ▶; Tang *et al.*, 2012 ▶, 2014 ▶).

It should be noted that the effect of increasing pressure on the structure of lipids and lipid assemblies is generally qualitatively similar to the effect of decreasing temperature (Brooks *et al.*, 2011 ▶). However, at a phase-transition boundary (where the free energy change, Δ*G*, for the transition is zero), this relationship can be quantified using the Clapeyron equation [equation (1)] to determine the pressure dependence of a lipid phase-transition temperature, *T*_t_

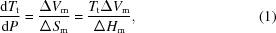
where Δ*S*_m_, Δ*H*_m_ and Δ*V*_m_ are the molar transition entropy, enthalpy and volume changes, respectively. These parameters can be measured at, or very near, atmospheric pressure, using differential scanning calorimetry (DSC) to determine *T*_t_, Δ*S*_m_ and Δ*H*_m_, and pressure perturbation calorimetry (PPC) to measure *T*_t_ and Δ*V*_m_. Δ*S*_m_ and Δ*V*_m_ can generally be assumed to be independent of pressure (or to have the same pressure dependence) up to around 200 MPa, and so the Clapeyron equation predicts a linear relationship between transition temperature and pressure.

### 2.2. Bilayer structure   

In addition to their structural role, a key function of biomembranes is to provide an active two-dimensional lipid matrix within which reactions can take place. The dynamic lateral organization and structure in these membranes are thought to play key roles in regulating a wide range of cell processes (Staubach & Hanisch, 2011 ▶; Bethani *et al.*, 2010 ▶) and pressure can play a key role in investigating this ordering.

As well as influencing the mesoscopic phase behaviour of lipids, pressure can cause more subtle changes in the structure of lipid bilayers and has a significant effect on the micromechanics of membranes. As described above, increasing pressure causes increased hydrocarbon chain ordering and, for flat lipid bilayers, this will tend to lead to an increase in the bilayer thickness, accompanied by a reduction in the area per hydrocarbon chain.

High pressures can cause single-component fluid lamellar bilayers, where the hydrocarbon chains are effectively molten, to undergo a phase transition to a lamellar gel structure (Cheng *et al.*, 1996 ▶; Shaw *et al.*, 2012 ▶), where the hydrocarbon chains are now fixed in specific lattice positions in an almost all-*trans* conformation, and dynamic high-pressure experiments have been used to probe the mechanism of fluid–gel phase transitions (Cheng & Caffrey, 1996 ▶). However, even pressure changes that are too small to induce gelling of a fluid bilayer tend to lead to bilayer swelling due to chain extension, with a change in bilayer thickness of less than 2 Å kbar^−1^.

**Figure 4:**
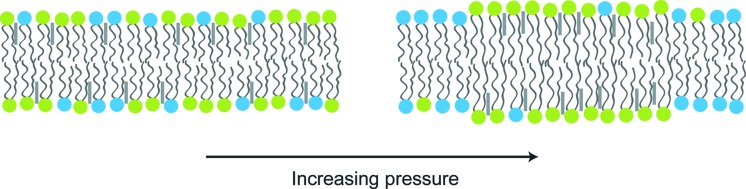
Pressure can drive separation between coexisting fluid phases in ternary lipid mixtures. Increasing pressure causes ordering of the lipid chains, which leads to association of the higher melting point lipids (green) and cholesterol (grey rods) to form liquid ordered domains, coexisting with liquid disordered domains formed primarily from the lower melting point lipid (blue).

As mentioned above, lateral structuring in biomembranes, and its effect on protein function and regulation, is thought to be a key property of cellular membranes. Model membranes have been used extensively to gain a valuable insight into lateral ordering in bilayers (Veatch *et al.*, 2004 ▶) and pressure is ideally suited to triggering these types of structuring due to the rapid characteristic timescales. In bilayers made from binary mixtures of lipids with different chain melting temperatures (and so, as shown by the Clapeyron equation, different chain melting pressures at a fixed temperature), pressure has been shown to induce phase separation between fluid and gel structures (Winter & Jeworrek, 2009 ▶). Ternary mixtures of a high-melting-point lipid, low-melting-temperature lipid and cholesterol can exhibit coexistence between two different fluid phases, liquid disordered domains (where the lipid hydrocarbon chains are molten, as in an L_α_ phase) and liquid ordered (L_o_) domains (Veatch *et al.*, 2004 ▶) (where the lipids exhibit fast diffusion within the bilayer but the hydrocarbon chains show a high degree of conformational ordering) (Fig. 4 ▶). It has recently been shown that pressure can be used to drive liquid–liquid phase separation (Nicolini *et al.*, 2006 ▶; Jeworrek *et al.*, 2008 ▶), and this coexistence can be probed using both small-angle X-ray diffraction and microscopy (Nicolini *et al.*, 2006 ▶; Tayebi *et al.*, 2012 ▶). There is currently a significant amount of ongoing work aimed at elucidating the biophysical parameters that determine the extent, stability and kinetics of model membrane structuring, and linking this to dynamic ordering in biological membranes. High-pressure and pressure-jump experiments are likely to be extremely important in unlocking the bottlenecks associated with rapid triggering of these types of structural change and have the potential to underpin a wide range of exciting dynamic membrane structural studies.

Changes in the structure of a membrane will inevitably cause changes in the micromechanical properties of the membrane. The fundamental parameters that underpin the micromechanics of a membrane are described by equation (2) for the curvature elastic energy, *g*_c_, of a lipid monolayer (Helfrich, 1973 ▶)
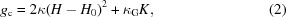
where *H* = 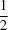
(*c*_1_ + *c*_2_) and *K* = *c*_1_ *c*_2_ are the average mean and Gaussian curvatures, respectively; *c*_1_ and *c*_2_ are the principal curvatures at a given point on the surface; *H*_0_ is the spontaneous mean curvature; and κ and κ_G_ are the mean and Gaussian curvature moduli, respectively. The mean curvature modulus describes the energetic cost of changing the mean curvature of a monolayer, whereas the Gaussian modulus represents the energy required to change the Gaussian curvature.

The corresponding parameters can be found for a bilayer: the preferred curvature *H*_0_ for a symmetrical bilayer must be zero, and the bilayer bending modulus κ^b^ is expected to be simply twice the monolayer modulus (Seddon & Templer, 1993 ▶). However, the expression for the bilayer Gaussian modulus, 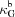, is more complex (Helfrich & Rennschuh, 1990 ▶)
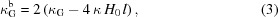
Here, all parameters on the right-hand side refer to a monolayer, including *l*, the monolayer thickness. κ^b^ and 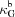 refer to the energetic cost of exactly the same physical deformations as described previously for the monolayer.

It has been suggested that pressure increases the monolayer bending modulus (Kawabata *et al.*, 2004 ▶) and it is expected that pressure will also increase the bending modulus of a bilayer (Shearman *et al.*, 2006 ▶), due to pressure-induced bilayer thickening as discussed earlier. It has been shown that pressure increases the monolayer spontaneous curvature, *H*_0_ (Winter *et al.*, 1999 ▶). However, it is worth noting that lipids which tend to form inverse structures have a negative spontaneous curvature, so pressure will tend to decrease the magnitude of this negative curvature. The observation that pressure can stabilize bicontinuous cubic lipid phases (Duesing *et al.*, 1997 ▶) which have a negative Gaussian curvature suggests that pressure also increases the bilayer Gaussian modulus, 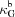 (thereby reducing the bilayer curvature elastic energy). However, for lipids which tend to form inverse phases, the effect of pressure on the monolayer Gaussian modulus is far less clear. In equation (3), pressure increases κ but decreases the magnitude of *H*_0_, which will tend to cancel out, making it difficult to predict the effect on κ_G_.

### 2.3. Lipid–protein assemblies   

While biological membranes were once thought to consist of active membrane proteins which are associated with a passive lipid structural matrix, it is now known that the protein concentrations can reach as high as 30 wt% in membranes, and both the proteins and lipids play a highly active role in cellular processes (Staubach & Hanisch, 2011 ▶; Bethani *et al.*, 2010 ▶).

Lipid–protein interactions have increasingly been recognized as being critical to a range of cellular processes and signalling events, and it is now recognized that lipids and membrane proteins must interact strongly both physically and chemically (Lee, 2003 ▶; Charalambous *et al.*, 2012 ▶). The overall structural response of biomembranes to external influences such as pressure is likely to result from the close coupling of changes in both the lipids and the proteins, and their interactions. There have so far been relatively few studies of the influence of pressure on model lipid–protein assemblies, but careful control of temperature and pressure has the potential to facilitate future investigation of the mechanisms and dynamics of lipid–protein co-structuring. Two key examples of the effect of pressure on lipid–protein structures are described below.

Incorporation of the small peripheral membrane protein cytochrome *c* into inverse bicontinuous cubic lipid phases formed from monoolein has been found to induce significant changes in the structural behaviour of the membrane (Lendermann & Winter, 2003 ▶). The incorporation of low concentrations of protein shifts the temperature and pressure phase boundaries for monoolein. At higher protein concentrations, the formation of a noncentrosymmetric cubic phase of space group *P*4_3_32 is observed. This structure is thought to be similar to that of the gyroid bicontinuous cubic phase, with one water channel replaced by inverse micelles at the junction points, and with one molecule of cytochrome *c* positioned in the centre of each inverse micelle, suggesting that the lipid–protein interaction drives an increase in the magnitude of the inverse membrane curvature. The pressure stability of this novel structure increases as the protein concentration is increased, which is attributed to attractive protein–lipid headgroup interactions. Pressure-jump X-ray experiments have been used to probe the kinetics of phase transitions in this system (Kraineva *et al.*, 2005 ▶) and they show significantly slower transition times than in pure monoolein. Again, this is likely to be due to the attractive lipid–protein interactions and the necessity for co-structuring of the two components.

Monoolein with the integral membrane protein bacterio­rhodopsin incorporated (Kulkarni *et al.*, 2013 ▶) also shows significantly different pressure–temperature structural behaviour to pure monoolein. Inclusion of the protein increases the pressure stability of the observed bicontinuous cubic structures relative to flat lamellar phases, again suggesting that the protein–lipid interactions present here favour inverse membrane curvature. Highly swollen bicontinuous cubic phases (with lattice parameters of over 200 Å) have been observed at high pressure in these mixtures, both at equilibrium and during pressure-jump X-ray diffraction experiments.

### 2.4. Pressure-jump kinetic experiments and structural transformation   

One of the significant advantages of pressure over other structure-change triggers such as temperature or composition variation is that pressure can be changed extremely quickly: in a number of high-pressure instruments, pressure jumps of several hundred MPa can be performed in 5 ms (Brooks *et al.*, 2010 ▶; Woenckhaus *et al.*, 2000 ▶), and in some cases pressure jumps can be performed on a sub-microsecond timescale (Dumont *et al.*, 2009 ▶) (see below for further discussion of high-pressure technology). Such rapid changes allow the thermodynamic trigger to be decoupled from many biomolecule and membrane assembly structure changes, allowing the out-of-equilibrium behaviour of fast structural transitions in these systems to be characterized.

Pressure jumps have been used to yield valuable information about the kinetics and intermediates involved in the structural transitions of a number of the systems discussed above (Kriechbaum *et al.*, 1993 ▶; Conn *et al.*, 2008 ▶; Jeworrek *et al.*, 2008 ▶; Kulkarni *et al.*, 2013 ▶). Recently, a significant advance has been made in quantitative modelling of lipid phase transitions with the development of a kinetic model for lipid structural transitions involving monolayer curvature change (Squires *et al.*, 2009 ▶). If a suitable kinetic model can be fitted to describe a structural transition, the rate at which the transition takes place can be related to the volume of activation, Δ*V*_a_

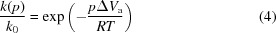
where *k*(*p*) and *k*_0_ are the rate constants at relative pressure *p* and atmospheric pressure, respectively, *R* is the universal gas constant and *T* is the temperature. The volume of activation can be interpreted using transition-state theory as the difference in volume between the transition state and the volume of the reactants at the same pressure. This can be thought of as an elastic barrier to transformation, in much the same way as the activation energy for a reaction is thought of as a thermal energetic barrier to a reaction.

## 3. High-pressure instrumentation   

As described above, pressure can play a key role in studying the structure of dynamic membrane assemblies and the biology that they underpin. To take full advantage of high-pressure technology, it is essential that it is coupled to fast structure probe techniques, and a great deal of work has been focused on linking high-pressure instrumentation with synchrotron X-ray diffraction and scattering facilities (Woenckhaus *et al.*, 2000 ▶; Ando *et al.*, 2008 ▶; Krywka *et al.*, 2008 ▶; Brooks *et al.*, 2010 ▶; Fourme *et al.*, 2012 ▶, 2011 ▶; Girard *et al.*, 2010 ▶). This has also facilitated significant advances in the accessibility of high-pressure instrumentation (Brooks *et al.*, 2010 ▶). In addition, high-pressure NMR (Bonev & Morrow, 1997*b* ▶; Peng & Jonas, 1992 ▶), optical microscopy (Nicolini *et al.*, 2006 ▶; Vass *et al.*, 2010 ▶) and spectroscopy (Schiewek *et al.*, 2007 ▶; Dumont *et al.*, 2009 ▶) have seen rapid development and provide highly complementary structural data to X-ray experiments.

### 3.1. High-pressure sample cells for small-angle X-ray scattering (SAXS)   

Small-angle X-ray diffraction is ideally suited to probing the structure of lyotropic lipid membrane assemblies, which are ordered on the nanoscale. Additionally, SAXS has been increasingly employed for studying the structures of proteins and protein assemblies (Petoukhov *et al.*, 2012 ▶; Tuukkanen & Svergun, 2014 ▶).

SAXS high-pressure sample cells have to be carefully designed to hold relatively large sample volumes (several microlitres) at high pressure, while allowing a wide scattering-angle range to be resolved. Within these constraints, SAXS pressure cells have been developed by a number of groups over more than 20 years (So *et al.*, 1992 ▶; Mencke *et al.*, 1993 ▶; Pressl *et al.*, 1997 ▶; Ando *et al.*, 2008 ▶; Krywka *et al.*, 2008 ▶; Brooks *et al.*, 2010 ▶) to address the need for fine pressure control of soft matter systems.

A significant landmark was reached with the development of a robust and versatile cell system by Woenckhaus *et al.* (2000 ▶). This system can perform both static and kinetic pressure experiments in the range 0–0.7 GPa (7 kbar) and −40–100°C. The pressure is generated and controlled *via* a water-filled hydraulic network that employs two air-operated valves to initiate the pressure jumps, one for jumps of increasing pressure and another for jumps of decreasing pressure. The hydraulic network approach allows pressure jumps to be performed in as little as 5 ms. The cell windows are 0.8 mm thick diamond, eliminating the risk of toxic dust formation associated with beryllium. The specifications of this pressure system have set a benchmark for more recent soft condensed matter pressure cells, and it has facilitated a wide range of high-pressure and pressure-jump experiments on lipid membranes (Conn *et al.*, 2008 ▶; Eisenblätter & Winter, 2006 ▶; Jeworrek *et al.*, 2008 ▶; Kraineva *et al.*, 2005 ▶; Tang *et al.*, 2012 ▶; Kulkarni *et al.*, 2013 ▶).

Recent developments have also been made in sample containment (Ando *et al.*, 2008 ▶), windows with low parasitic scatter (Wang *et al.*, 2012 ▶) and sample-loading ports (Krywka *et al.*, 2008 ▶). The development of a dedicated sample-loading port (in contrast with previous cells, which generally required samples to be loaded through one of the X-ray window ports) has the significant advantage of allowing accurate subtraction of background scattering due to the X-ray windows.

We have recently developed a high-pressure SAXS system based at beamline I22, Diamond Light Source, UK (Brooks *et al.*, 2010 ▶). Static and millisecond pressure-jump experiments can be carried out in the range 0.1–500 MPa and between −20 and 120°C. The system is fully automated, integrated with the beamline and available to all users of I22, which opens up high-pressure technology to an extremely wide user base.

### 3.2. Diamond anvil cells (DACs)   

Diamond anvil cells (Katrusiak, 2008 ▶) are routinely employed for high-pressure experiments requiring pressure up to 100 GPa (10^5^ bar). They consist of two opposing anvils which compress a sample held in a metal gasket. The simplest DACs apply a relatively small pressure to the diamonds *via* a screw system, and this pressure is intensified by the shape of the diamond and applied to the sample. Significant advances have recently been made in the design of DACs, particularly with the development of gas-membrane-driven cells, where a gas-filled ‘balloon’ applies the initial pressure, instead of a screw-driven brace. This has several advantages, including remote operation, the ability to apply small controlled pressure increments, and higher achievable pressures due to the absence of screw friction. The internal volume of a DAC is very small and so pressure measurements are usually made by placing a small ruby or α-quartz crystal in the sample and measuring the position of the *R*_1_ ruby fluorescence maximum (Piermarini *et al.*, 1975 ▶) or quartz IR vibration (Wong *et al.*, 1985 ▶), which shift with pressure, offering a resolution of around 20 MPa (Czeslik *et al.*, 1998 ▶). There has been significant interest recently in developing alternative pressure transducers for use in DACs with increased resolution (Oger *et al.*, 2006 ▶; Picard *et al.*, 2006 ▶).

DACs have proved extremely valuable in studying protein behaviour at high pressure (Silva *et al.*, 2001 ▶). While the pressures accessible with DACs are often significantly higher than required to study membrane structure changes, lipid studies have been carried out at up to 2 GPa (Czeslik *et al.*, 1998 ▶; Reis & Winter, 1998 ▶) and recent advances, particularly in pressure detection (Picard *et al.*, 2006 ▶) and control (Oger *et al.*, 2006 ▶), may open up new avenues in very high-pressure lipid and membrane research. A significant advantage of DACs over the soft matter SAXS cells described above is that far wider diffraction angles can be resolved, since the diamond anvils are relatively X-ray transparent. DAC experiments have proved particularly valuable in macromolecular crystallography (Fourme *et al.*, 2012 ▶) and this technique is now widely available at synchrotron beamlines offering extreme conditions (Fourme *et al.*, 2011 ▶).

### 3.3. Complementary high-pressure structure probe techniques   

#### 3.3.1. High-pressure NMR   

Several high-pressure NMR systems have been reported (Fourme *et al.*, 2012 ▶). However, the high-pressure NMR probe developed by Bonev & Morrow (1997*b* ▶) was designed specifically for use with soft matter and has been used extensively to study model membrane samples (Bonev & Morrow, 1997*a* ▶; Fiech *et al.*, 1998 ▶). This probe allows studies up to 300 MPa at temperatures between −20 and 100°C.

#### 3.3.2. High-pressure optical microscopy   

There have been exciting recent developments in the design and use of high-pressure optical microscopy systems, allowing bright-field (Frey *et al.*, 2006 ▶; Nishiyama & Kojima, 2012 ▶), polarizing (Reck *et al.*, 1998 ▶), fluorescence (Nicolini *et al.*, 2006 ▶; Vass *et al.*, 2010 ▶; Nishiyama *et al.*, 2009 ▶) and single-molecule (Vass *et al.*, 2013 ▶) microscopy, at pressures as high as 700 MPa (Vass *et al.*, 2010 ▶). A number of these cells have again been based on similar design principles to the soft matter SAXS cells described above (Reck *et al.*, 1998 ▶). However, there has also been a very successful optical pressure cell constructed from narrow-bore fused silica capillary tubing (Nicolini *et al.*, 2006 ▶). A significant consideration for high-resolution microscopy is allowing close access of the microscope objective lens to the sample while maintaining the pressure stability of the cell. The working distance of objective lenses falls as their magnification increases, but magnification of up to 40× has been achieved using a metal body/window type cell (Frey *et al.*, 2006 ▶; Nishiyama *et al.*, 2009 ▶), and 63× using the capillary tube system mentioned above (Nicolini *et al.*, 2006 ▶).

#### 3.3.3. Spectroscopy pressure cells   

A number of high-pressure soft matter cells have been developed for use with Fourier-transform IR (FT–IR) spectroscopy, following a similar design to the soft matter SAXS cells above (Schiewek *et al.*, 2007 ▶) and using a hydraulic network to generate pressure. These have been used at static pressures up to 600 MPa and for pressure jumps (Schiewek *et al.*, 2007 ▶). In addition, DACs have been used successfully for FT–IR experiments (Czeslik *et al.*, 1998 ▶).

The speed of pressure jumps generated by a hydraulic valve system as described above is limited to around 5 ms by the time required to open the pressure-jump valve. While this is considerably faster than many biomolecular transformations, some structure changes (particularly protein structure changes) can occur on a shorter timescale, which has driven the development of faster pressure-jump technology.

Spectroscopy pressure cells have been developed with a piezoelectric stack piston built into the cell body (Pearson *et al.*, 2002 ▶) that can generate extremely fast pressure jumps. Although the movement of the piston limits the pressure that can be reached, jumps of up to 20 MPa can be performed in 150 µs while probing the sample by absorption or fluorescence spectroscopy.

Burst diaphragms have been used for many years to generate rapid downward pressure jumps (Davis & Gutfreund, 1976 ▶) but there has recently been a significant advance in burst-membrane technology with the development of an electrically ruptured burst diaphragm (Dumont *et al.*, 2009 ▶). This allows the rupture to be induced at an accurately set pressure, and downward pressure jumps of up to 250 MPa can be performed in less than 700 ns, providing the fastest pressure jumps currently available.

## 4. Concluding remarks   

High pressure has proved to be an extremely powerful biophysical tool for studying the structural behaviour of membrane assemblies. It has facilitated investigation of the mechanisms of large-scale structure changes in lipid mesophases, the kinetics of phase separation and ordering with bilayers, and the stability of lipid–protein assemblies, amongst a wide variety of other experiments. Developments in high-pressure instrumentation continue to widen the scope of pressure technology, both in terms of the samples that it can be applied to and the groups that can make use of it. With exciting new developments such as ultra-fast pressure-jump technology and high-resolution high-pressure microscopy, there will clearly be a wide range of experiments in the near future which will provide ever greater insight into the structure and function of dynamic biological membranes.
